# Resuscitative endovascular balloon occlusion of the aorta versus aortic cross clamping among patients with critical trauma: a nationwide cohort study in Japan

**DOI:** 10.1186/s13054-016-1577-x

**Published:** 2016-12-15

**Authors:** Toshikazu Abe, Masatoshi Uchida, Isao Nagata, Daizoh Saitoh, Nanako Tamiya

**Affiliations:** 1 Department of Health Services Research, Faculty of Medicine, University of Tsukuba, 1-1-1, Tennodai, Tsukuba, 305-8577 Japan; 2 Department of Emergency Medicine, Tsukuba Medical Center Hospital, 1-3-1, Amakubo, Tsukuba, 305-8558 Japan; 3 Department of Traumatology and Emergency Medicine, National Defense Medical College, 3-2, Namiki, Tokorozawa, Saitama 359-8513 Japan

## Abstract

**Background:**

Measures of aortic occlusion (AO) for resuscitation in patients with severe torso trauma remain controversial. Our aim was to characterize the current use of resuscitative endovascular balloon occlusion of the aorta (REBOA) and resuscitative open aortic cross-clamping (ACC), and to evaluate whether REBOA should be an alternative method to resuscitative open ACC.

**Methods:**

This study was a retrospective cohort study between 2004 and 2013 from a nationwide trauma registry in Japan. Participants were selected who underwent either REBOA or ACC. Their characteristics, interventions, and outcomes were analyzed to compare REBOA and ACC directly. The primary outcome was in-hospital mortality and the secondary outcome was mortality in the emergency department. Logistic regression analysis was performed to compare the outcomes between REBOA and ACC with adjustment for severity; 1:1 propensity score matching was also performed.

**Results:**

Of the 159,157 trauma patients, 903 were eligible based on the selection criteria. Overall, 405/607 patients (67%) who had REBOA died compared to 210/233 patients (90%) who had ACC. Patients with REBOA had higher revised trauma score (RTS) (mean ± SD, 5.2 ± 2.0 vs. 4.2 ± 2.2; *P* < 0.001) but higher Injury Severity Score (ISS) (median (interquartile); 34 (25) vs. 34 (20); *P* < 0.001), and higher probability of survival (0.43 ± 0.36 vs. 0.27 ± 0.30; *P* < 0.001) compared to those with ACC. REBOA had an odds ratio (OR) for in-hospital mortality of 0.309 (95% confidence interval (CI) = 0.190–0.502) adjusting for trauma and injury severity score using a logistic regression model (*n* = 903). Similar associations were observed adjusting for RTS (OR = 0.224; 95% CI = 0.129–0.700) or adjusting for ISS (OR, 0.188; 95% CI, 0.116 to 0.303). In the propensity score-matched cohort (*n* = 304), REBOA was associated with lower mortality compared to ACC (OR, 0.261; 95% CI, 0.130 to 0.523). Patients with REBOA had less severe chest complications than those with ACC (Abbreviated Injury Scale thorax, 3.8 ± 0.8 vs. 4.2 ± 0.8; *P* < 0.001), although physiological severity and backgrounds were similar in this population.

**Conclusions:**

Patients who underwent AO had a high mortality. REBOA might be a favorable alternative method to resuscitative ACC for severe torso trauma although some indication bias could still remain. Further studies are needed to elucidate optimal indications.

## Background

Bleeding control is a critical strategy in the management of severe trauma patients. Aortic occlusion (AO) is a standard initial procedure to control blood loss in severe torso trauma patients which buys time for a more definitive treatment. Open aortic cross-clamping (ACC), established as just such a definitive approach, has been traditionally accomplished via emergent thoracotomy or as an initial step during laparotomy [1]. However, ACC for resuscitation in critical trauma patients remains controversial because of a very high mortality rate. In actuality, open ACC might suffer from a negative perception because it might be used in patients already beyond saving, thereby driving up the mortality rate. Recently, resuscitative endovascular balloon occlusion of the aorta (REBOA) has been used as an alternative method of ACC. REBOA has been previously described as useful for hemorrhagic shock in cases of ruptured abdominal aortic aneurysm [2], gastrointestinal bleeding [3], and in postpartum hemorrhage [4]. As they are useful in solving multiple problems, endovascular approaches such as trans-catheter arterial embolization (TAE) should become more widely used in trauma settings. However, there is a dearth of clinical reports with adequate sample size and situations on which to base recommendations [5, 6]. There are a few reports to show favorable outcomes of REBOA compared with ACC [1] but no concrete indications of REBOA or ACC efficacy exist at the time of this report. To this end, our aim was to analyze the present situation of REBOA and ACC usage with nationwide trauma registry data and to then evaluate as to whether or not REBOA should be deemed a preferential alternative to resuscitative ACC.

## Methods

### Study designs

We conducted a retrospective cohort study using registered data from the Japan Trauma Data Bank (JTDB) to compared characteristics and outcomes between REBOA and ACC.

### Data collection

Data were obtained from the JTDB, a nationwide trauma registry established in 2003 and authorized and maintained by the Japanese Association for the Surgery of Trauma and the Japanese Association for Acute Medicine to improve and assure the quality of trauma care in Japan [7]. During the study period, a total of 234 hospitals including 95% of tertiary emergency medical centers in Japan participated in the JTDB [7]. The JTDB collected variables about patients and hospitals such as patient demographics, comorbidities, injury type, mechanism, vital signs, Abbreviated Injury Scale (AIS) score, Injury Severity Score (ISS), pre-hospital treatment, in-hospital procedures, and in-hospital and emergency department (ED) mortality [7].

REBOA has recently found use as a general technique across major emergency centers in Japan. Due to the limitations of the JTDB as being a general, total trauma registry and not a REBOA-specific database, we were unable to glean information on imaging, access, and balloon zone placement specifics. Although details of REBOA in those three areas depend mainly on local facilities and expertise, in our specific cases REBOA access is typically accomplished through a common femoral artery and the balloon insertion follows a blind approach.

### Patient selection

**Fig. 1:**
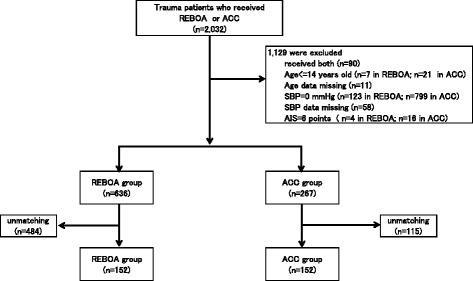
Flow chart of patients included in this study. *AIS* Abbreviated Injury Scale, *ACC* aortic cross-clamping, *REBOA* Resuscitative endovascular balloon occlusion of the aorta, *SBP* systolic blood pressure

The study inclusion criteria were the presence of critical trauma and reception of either REBOA or ACC. We excluded patients who had received both REBOA and ACC. Also, we excluded subjects younger than 14 years old or those with age data missing. Patients with cardiopulmonary arrest on arrival at the ED (systolic blood pressure of 0 mm Hg or data missing on arrival) or with an AIS score of 6 (i.e., non-survivable injury) for any region were also excluded. Figure 1 shows participant selection data from this study.

### Study endpoints

The primary intervention was either REBOA or ACC. Intervention strategies were solely dependent on the individual decisions of ED physicians. The primary outcome of this study was in-hospital mortality and the secondary outcome was mortality in the ED.

### Statistical analysis

To report the characteristics of participants, firstly we used the Shapiro-Wilk test to identify normality of each variable. However, there was some skew in almost all the variables. We then decided whether to use the mean ± standard deviation (SD) or median (interquartile range (IQR)) for plot figures of each variable, depending on the previous reporting style. To assess the independent effects of REBOA compared with those of ACC, outcomes were evaluated by analytical models—standard logistic regression and a logistic regression incorporating the results of propensity score matching. Since the number of survivors was very small in this study population, we chose a few covariates for the standard logistic regression analyses. Within the standard logistic regression analyses, we conducted three adjustment models: a revised trauma score (RTS)-adjusted model, an ISS-adjusted model, and a trauma and injury severity score (TRISS)-adjusted model. Together, these three standard logistic regression models, coupled with a propensity score-matched model, provide a robust method for statistically reliable analysis.

### Propensity score

Because the use of REBOA or ACC was not randomly assigned, a formal causal inference is not possible. Therefore, a logistic regression analysis was used to estimate propensity scores (PSs) to predict usage of REBOA or ACC from the available predictors. These variables were age, gender, mechanism of injury, cause of injury, transport type, pre-hospital treatment, vital signs at ED, and ISS, which would reflect patient wound severity. Treatments after ED arrival (e.g., blood transfusion, cardiopulmonary resuscitation (CPR), operations) were not included in the PS derivation process because they were performed after usage of REBOA or ACC. Severity scoring systems without ISS (e.g., RTS, TRISS) were not included in the PS because in them were many of the same components that had already been included in the PS, such as vital signs. Propensity score matching extracted 1:1 matched pairs of subjects who received REBOA or ACC based on an averaged PS. The absolute standardized difference of variables for the PS estimation was used to assess the match balance. An absolute standardized difference of less than 0.2 was generally considered as an acceptable match balance between the groups.

The two-sided significance level for all tests was *P* < 0.05. All analyses were performed using SPSS software, version 21.0 (IBM, Armonk, NY, USA).

## Results

A total of 159,157 trauma patients were registered in the JTDB from 1 January 2004 to 31 December 2013. Of these, 2032 patients with trauma were included in this study because they received REBOA or ACC. Cases were excluded if they received both REBOA and ACC (*n* = 90), were below the cutoff age (*n* = 28), had age data missing (*n* = 11), if they had already died (*n* = 922), had SBP data missing (*n* = 58), or if they did not have a chance to survive (i.e., AIS = 6) (*n* = 20). Thus, 903 patients were included in the first round of calculations. After PS matching, 304 patients were included in a second round of analysis (Fig. 1).

**Fig. 2:**
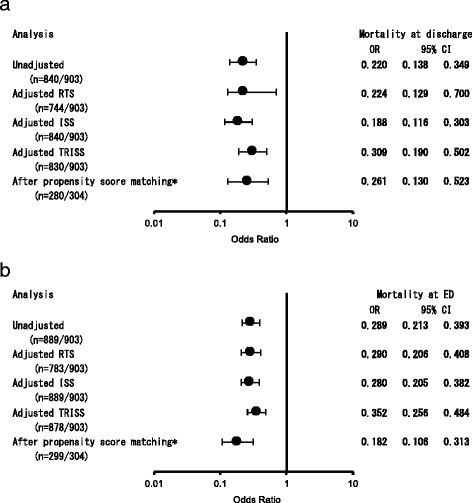
Comparison of the mortality of REBOA versus ACC at discharge (**a**) and in the emergency department (*ED*) (**b**). *The covariates used to estimate the propensity score were age, gender, mechanism of injury, cause of injury, transport type, prehospital treatment, vital signs at ED, and Injury Severity Score (*ISS*). *CI* confidence interval, *OR* odds ratio, *RTS* revised trauma score, *TRISS* trauma and injury severity score

Table 1 shows the characteristics of critical patients with trauma who received either REBOA or ACC. The mean age was 53.7 ± 21.2 years; 611/903 (67.7%) were male. Blunt trauma was common (838/895; 93.6%). Mean RTS was 4.94 ± 2.08. The RTS in REBOA cases was significantly higher than in ACC cases. Median (interquartile) ISS was 34 (20). ISS in REBOA cases were also more severe than that in ACC cases. However, TRISS was higher in REBOA cases than in ACC cases. Also, 153/636 (24%) patients who were REBOA cases received TAE compared to 18/267 (6.7%) who were ACC cases. Table 2 demonstrates outcome comparisons between REBOA and ACC. In-hospital mortality was 405/607 (67%) in REBOA and 210 /233 (90%) in ACC. ED mortality was 137/625 (22%) in REBOA and 130/264 (49%) in ACC. Figure 2 shows a comparison of the mortality between REBOA and ACC (*n* = 903). Patients who underwent REBOA had a significantly lower in-hospital mortality than those who underwent ACC as shown by adjusted RTS (odds ratio (OR) = 0.224; 95% confidence interval (CI) = 0.129–0.700), ISS (OR = 0.188; 95% CI = 0.116–0.303), or TRISS (OR = 0.309; 95% CI = 0.190–0.502), respectively. After PS matching (*n* = 304), in-hospital mortality was 106/146 (73%) in REBOA and 122/134 (91%) in ACC, and ED mortality was 24/149 (16%) in REBOA and 77/150 (51%) in ACC. Thus, mortality in the REBOA patients was lower than that of ACC (OR = 0.261; 95% CI = 0.130–0.523 at discharge; OR = 0.182; 95% CI = 0.106–0.313 at ED). Table 3 lists the baseline characteristics of PS-matched patients (*n* = 304). There was no significant difference between REBOA and ACC in RTS (mean ± SD; 4.8 ± 2.0 vs 4.7 ± 2.1; *P* = 0.631), ISS (median (interquartile); 34 (23) vs 36 (20), *P* = 0.341), and TRISS (mean ± SD; 0.45 ± 0.35 vs 0.39 ± 0.31, *P* = 0.115). However, the AIS of the thorax was significantly lower in REBOA cases than in ACC cases (3.8 ± 0.8 vs 4.2 ± 0.8, *P* < 0.001). Thoracotomy at initial evaluation was also less frequent in REBOA cases than in ACC cases. On the other hand, patients with REBOA underwent angiography of the abdomen and pelvis, including TAE, more often than those with ACC.

**Table 1:** Characteristic of critical patients with trauma who had either REBOA or ACC

	REBOA (*n* = 636)	ACC (*n* = 267)	*P* value	Missing
Age (years)	52.5 ± 21.2	56.7 ± 21.1	0.007	0
Gender (male)	417/636 (66%)	194/267 (73%)	0.043	0
Onset year				0
2004–2008	218/636 (34%)	70/267 (26%)		
2009–2013	418/636 (66%)	197/267 (74%)		
Mechanism of injury (blunt vs. penetrating)	591/630 (94%)	247/265 (93%)	0.765	8
Cause of injury			0.754	31
Accident	429/618 (69%)	187/254 (74%)		
Suicide	127/618 (21%)	44/254 (17%)		
Assault	20/618 (32%)	6/254 (2.4%)		
Workplace injuries	39/618 (6.3%)	16/254 (6.3%)		
Other	1/618 (0.2%)	1/254 (0.4%)		
Transport type			0.008	27
Ambulance	514/617 (83%)	194/259(75%)		
Ambulance with physician	26/617 (4.2%)	24/259 (9.3%)		
Helicopter with physician	73/617 (12%)	40/259 (15%)		
Other	4/617 (0.6%)	1/259 (0.4%)		
Vital signs at prehospital				
SBP	101 (42)	105 (40)	0.42	358
HR	97 (37)	100 (43)	0.76	163
RR	24 (10)	25 (10)	0.445	232
Vital signs at emergency department				
GCS value	10(12)	5(8)	<0.001	21
SBP	89 (46)	87 (45)	<0.001	0
HR	102 (36)	106 (52)	0.181	14
RR	25 (10)	24 (15)	<0.001	99
RTS	5.2 ± 2.0	4.2 ± 2.2	<0.001	107
AIS				0
Head (*n* = 382)	3.6 ± 1.2	3.3 ± 1.1	0.101	
Face (*n* = 167)	1.6 ± 0.7	1.8 ± 1.3	0.274	
Neck (*n* = 14)	2.4 ± 1.3	1.5 ± 0.8	0.178	
Thorax (*n* = 593)	3.8 ± 0.9	4.3 ± 1.1	<0.001	
Abdomen and pelvis (*n* = 580)	3.6 ± 1.1	3.8 ± 1.5	0.143	
Spine (*n* = 187)	2.5 ± 1.1	2.6 ± 0.9	0.959	
Upper extremity (*n* = 209)	2.0 ± 0.6	2.1 ± 0.6	0.284	
Lower extremity (*n* = 558)	3.7 ± 1.3	3.7 ± 1.2	0.702	
Others (*n* = 39)	1.1 ± 0.4	1.2 ± 0.4	0.701	
ISS	34 (25)	34 (20)	<0.001	0
TRISS (probability of survival)	0.43 ± 0.36	0.27 ± 0.30	<0.001	12
Prehospital treatment				
Airway protection maneuver	53/636 (8.3%)	41/267 (15%)	0.003	0
Intubation	44/636 (6.9%)	35/267 (13%)	0.004	0
Intravenous fluid	55/636 (8.6%)	25/267 (9.3%)	0.703	0
FAST			0.013	29
Positive	359/614 (59%)	133/260(51%)		
Negative	233/614 (38%)	107/260 (41%)		
Not conducted	22/614 (3.6%)	20/260 (7.7%)		
Blood transfusion	542/636 (85%)	197/267 (74%)	<0.001	0
CPR				
ERT with CPR	71/636 (11%)	216/267 (81%)	<0.001	0
Closed CPR	141/636 (22%)	92/267 (35%)	<0.001	0
Operation at initial evaluation				
Craniotomy	19/636 (3.0%)	1/267 (0.4%)	0.012	0
Craterization	17/636 (2.7%)	3/267 (1.2%)	0.215	0
Thoracotomy	70/636 (11%)	160/267 (60%)	<0.001	0
Laparotomy	301/636 (47%)	99/267 (37%)	0.005	0
Angiography				
Chest	29/636 (4.6%)	7/267 (2.6%)	0.196	0
Abdomen	156/636 (25%)	15/267 (5.6%)	<0.001	0
Pelvis	151/636 (24%)	22/267 (8.2%)	<0.001	0
TAE (all)	153/636 (24%)	18/267 (6.7%)	<0.001	0

All categorical variables are shown as *n* (%); continuous variables are shown as mean ± standard deviation or median (interquartile)

*ACC* aortic cross-clamping, *AIS* Abbreviated Injury Score, *CPR* cardiopulmonary resuscitation, *ERT* Emergency resuscitative thoracotomy, *FAST* Focused assessment with sonography for trauma, *GCS* Glasgow Coma Scale, *HR* heart rate, *ISS* Injury Severity Score, *REBOA* resuscitative endovascular balloon occlusion of the aorta, *RR* Respiratory rate, *RTS* revised trauma score, *SBP* systolic blood pressure, *TAE* trans-catheter arterial embolization, *TRISS* trauma and injury severity score

**Table 2:** Outcome comparisons between REBOA and ACC

	REBOA (*n* = 636)	ACC (*n* = 267)	*P* value
Disposition at discharge			<0.001*
Died (in-hospital mortality)	405/607 (67%)	210/233 (90%)	
Transferred	118/607 (19%)	11/233 (1.8%)	
Home	83/607 (14%)	12/233 (2.0%)	
Other	1/607 (0.1%)	0/233 (0.0%)	
Disposition at ED			<0.001*
Died (ED mortality)	137/625 (22%)	130/264 (49%)	
CU admission	472/625 (76%)	129/264 (49%)	
Ward admission	137/625 (22%)	4/264 (1.5%)	
Other	5/625 (1.8%)	1/264 (0.4%)	

The variables are shown as *n* (%)

*ACC* aortic cross clamping, *ED* emergency department, *ICU* intensive care unit, *REBOA* resuscitative endovascular balloon occlusion of the aorta

*Chi-square test

**Table 3:** Baseline characteristic in propensity score-matched patients with severe trauma*

	REBOA (*n* = 152)	ACC (*n* = 152)	SD	*P* value	Missing
Age (years)	52.8 ± 21.0	54.8 ± 22.1	0.09	0.421	0
Gender (male)	111/152 (73%)	101/152(66%)	0.14	0.261	0
Mechanism of injury (blunt vs. penetrating)	142/152 (93%)	141/152(93%)	0.03	1.000	0
Cause of injury				0.726	0
Accident	104 (68%)	109 (72%)	0.07		
Suicide	28 (18%)	29 (19%)	0.02		
Assault	6 (3.9%)	5 (3.3%)	0.04		
Workplace injuries	14(92%)	9 (5.9%)	0.13		
Other	0(0%)	0 (0%)	0.00		
Transport type				0.542	0
Ambulance	124/152 (82%)	123/152(81%)	0.02		
Ambulance with physician	6/152 (3.9%)	10/152 (6.6%)	0.12		
Helicopter with physician	22/152 (15%)	19/152 (13%)	0.06		
Other	0/152 (0%)	0/152(0%)	0.00		
Vital signs at emergency department					
GCS value	8(10)	8(10)	0.02	0.909	0
SBP	77.5 (64)	73.5 (64)	0.02	0.421	0
HR	108.0 (39)	109.5(52)	0.05	0.687	0
RR	25 (10)	24 (14)	0.08	0.499	0
RTS	4.8 ± 2.0	4.7 ± 2.1		0.631	0
ISS	34 (23)	36 (20)	0.11	0.341	0
TRISS (probability of survival)	0.45 ± 0.35	0.39 ± 0.31		0.115	0
Prehospital treatment					
Airway protection maneuver	12/152 (7.9%)	11/152 (7.2%)	0.03	1.000	0
Intubation	9/152 (5.9%)	13/152 (8.6%)	0.10	0.508	0
Intravenous fluid	9/152 (5.9%)	14/152 (9.2%)	0.13	0.386	0
FAST				0.232	6
Positive	91/148 (62%)	78/150(52%)			
Negative	52/148 (35%)	64/150(43%)			
Not conducted	5/148 (3.4%)	8/150 (5.3%)			
Blood transfusion	135/152 (89%)	121/152(80%)		0.04	0
AIS					0
Head	3.8 ± 1.3	3.4 ± 1.1		0.047	
Face	1.7 ± 0.8	1.3 ± 0.5		0.036	
Neck	1.3 ± 0.6	1.3 ± 0.5		0.846	
Thorax	3.8 ± 0.8	4.2 ± 0.8		<0.001	
Abdomen and pelvis	3.5 ± 1.0	3.8 ± 1.4		0.112	
Spine	2.6 ± 0.9	2.6 ± 1.0		0.812	
Upper extremity	2.1 ± 0.7	2.1 ± 0.6		0.833	
Lower extremity	3.6 ± 1.4	3.6 ± 1.3		0.904	
Other	1.0 ± 0.0	1.3 ± 0.5		0.172	
Operation at initial evaluation					
Craniotomy	3/152 (2.0%)	0/152(0%)		0.248	0
Craterization	4/152 (2.6%)	1/152 (0.7%)		0.371	0
Thoracotomy	20/152 (13%)	92/152(61%)		<0.001	0
Laparotomy	79/152 (52%)	68/152(45%)		0.251	0
Angiography					
Chest	2/152 (1.3%)	5/152 (3.3%)		0.448	0
Abdomen	27/152 (18%)	10/152 (6.6%)		0.004	0
Pelvis	33/152 (22%)	15/152 (9.9%)		0.007	0
TAE (all)	29/152 (19%)	11/152 (7.2%)		0.004	0
CPR					
ERT with CPR	20/152 (13%)	125/152 (82%)		<0.001	0
Closed CPR	33/152 (22%)	53/152(35%)		0.015	0

All categorical variables are shown as *n* (%); continuous variables are shown as mean ± standard deviation or median (interquartile)

*ACC* aortic cross-clamping, *AIS* Abbreviated Injury Score, *CPR* cardiopulmonary resuscitation, *ERT* Emergency resuscitative thoracotomy, *FAST* Focused assessment with sonography for trauma, *GCS* Glasgow Coma Scale, *HR* heart rate, *ISS* Injury Severity Score, *REBOA* resuscitative endovascular balloon occlusion of the aorta, *RR* Respiratory rate, *RTS* revised trauma score, *SBP* systolic blood pressure, *SD* standardized difference, *TAE* trans-catheter arterial embolization, *TRISS* trauma and injury severity score

*The covariates used to estimate the propensity score were age, gender, mechanism of injury, cause of injury, transport type, prehospital treatment, vital signs at the emergency department, and ISS

## Discussion

### Brief summary

This study investigated the current usage of REBOA and ACC using a large, nationwide trauma database in Japan. Mortality rates in patients requiring AO was discovered to be very high but this is attributed to the usage of ACC on patients who cannot be saved, skewing mortality out of favor with ACC. We also analyzed outcomes for patients after receiving either REBOA or ACC after adjusting for patient trauma severity. Robust analyses of the adjusted data showed that REBOA was associated with significantly reduced in-hospital mortality compared with ACC. However, due to differences in associated procedures between REBOA (e.g., increased need for angiography) and ACC (e.g., thoracotomy), there should be some consideration given to choosing either intervention.

### Comparison with previous studies

To our knowledge, our current study is one of the largest cohort studies describing the use of REBOA [8]. REBOA has recently found use as a general technique across major emergency centers in Japan. The highest density of potential REBOA patients is also seen at major trauma centers in England and Wales, although the number of patients in whom REBOA was utilized is small [9]. In fact, a review of the potential use of REBOA in exsanguinating hemorrhage cases in the US suggested that this new technique should be thoroughly evaluated for broad use, but the literature currently suffers from a dearth of human studies on REBOA [10]. Although our observational study admittedly had some selection bias, we feel that our results will nonetheless become an important part of the foundation of literature supporting the evaluation of global REBOA use.

Previous studies regarding REBOA usage have been limited in size and scope, and have shown ambiguous results. For example, although previous single-center cohort studies mentioned the utility of REBOA for massive pelvic bleeding cases that could still be imaged by angiography [5, 6], another large, retrospective cohort study cautioned against REBOA usage for patients who had emergency surgery or transcatheter embolization [7, 11]. Yet another single-center cohort study also reported on the feasibility and safety of REBOA for a non-compressive torso injury (pelvic fracture or hemoperitoneum) [12], but contrasting studies also reported that REBOA usage was associated with a higher mortality compared with non-REBOA usage in JTDB [7, 11]. However, to objectively evaluate these reports, a thorough knowledge of the Japanese trauma care system is required. For example, most Japanese emergency departments see few in-house trauma surgeries, see fewer trauma cases overall, and mostly deal with older patients and age-related maladies [13]. REBOA usage, in this context, may signal “last ditch” efforts [11]. However, our results from the same database show an incongruent outcome even though our study population and comparisons are different. Previous reports have indicated REBOA usage as a last resort in the most severe trauma cases, but only one multicenter, prospective observational study (Aortic Occlusion for Resuscitation in Trauma and Acute Care Surgery (AORTA) registry) [1] has looked at direct comparisons with ACC, which is also used in the most severe cases, and found REBOA to be beneficial. As the severity criteria for both REBOA and ACC are similar, it is reasonable to compare those outcomes directly and our reports findings strengthen the conclusion of DuBose and colleagues [1], and show a promising consistency in results.

### Possible explanations and implications

AO was used on 2032 patients in our database. However, 799 patients with ACC were excluded from analysis because of pre-hospital cardiopulmonary arrest. Although we controlled for this in our study, the differences between REBOA and ACC in general need more context for accurate interpretation. In Japan, ACC currently seems to be a preferred intervention tactic in non-survivable injury cases and this differs from other countries, possibly making Japanese ACC-related mortality rates non-indicative of actual outcomes [1, 9, 13]. In addition, other patient characteristics such as better Glasgow Coma Scale (GCS) scores in cases where REBOA was used versus ACC cases (with more severe GCS scores) may also skew results. This raises the issue of snap decisions by ED physicians to choose rapid thoracotomy over REBOA because ACC would be more frequently chosen in cases with worse GCS scores. A key point to keep in mind, however is that although the probability of survival (TRISS) for REBOA was better than that of ACC, it is still no guarantee of success in severe cases (0.43 ± 0.36). Furthermore, REBOA patients who die might count as preventable, but ACC deaths with TRISS scores indicating unsurvivable injuries (0.27 ± 0.30) would be counted as non-preventable. This might not be seen as a negative even though the mortality of ACC patients was very high (90%) and might be related to more severe complications in the thorax. These issues highlight the nuances necessary to objectively interpret the data, as both REBOA and ACC have complicating factors. Survival rates at ED of 78% and 51% in REBOA and ACC, respectively, indicate that both can serve a role in trauma treatment. However, it is important to keep in mind that these procedures are not panaceas; only 14% (83/607) of REBOA patients and 2.0% (12/233) of ACC patients could leave the hospital and go home. This emphasizes the difficulty of AO in clinical practice. This is especially evident in Japan as our mortality was slightly higher than other countries [1, 9, 13]. As Japan’s prevalence of penetrating trauma is quite low (6.4%), survival probabilities may follow suit. However, taking into consideration the variability in study populations and institutional skill, a general trend in the same direction can be seen with our results versus those of other countries.

Trauma severity in ACC cases versus REBOA cases were controlled for with sensitivity analyses as seen in Fig. 2, but REBOA usage showed a clear survival benefit. PS matching was also used to control for insufficiency of adjustment and the tendency of results was the same among PS-matched patients. We found that PS matching was one of the best methods to control confounders in this prevalence and mortality. Again, direct comparisons between REBOA and ACC were conducted after PS matching because of current interest in the possibility of shifting the ACC paradigm to REBOA [10]. Table 3 shows the precision of our PS matching methodology. However, covariate differences where we did not use PS (although we did properly control for physiological severity and backgrounds) unavoidably resulted in an inability to match anatomical severity. This might be classified as an indication bias, but we feel that our analysis shows the real utility of AO.

Taken together, we feel that these results should be made part of the body of knowledge that physicians consult in the decision tree of AO. Accordingly, REBOA would conceivably be used more often as a solo abdominal trauma option even though there are no formal criteria for AO treatment utility. Still, this does not necessarily mean that all AO cases would shift from ACC to REBOA. In reality, choosing ACC for severe abdominal trauma patients who present no chest trauma is a difficult choice for ED physicians who may not have enough thoracotomy experience. This is especially important because of recent reports on poor outcomes of emergency thoracotomies after abdominal exsanguination, adding to the reputation of ACC as a “last ditch” effort [13, 14]. However, to find the best position of an occlusion balloon with a blind approach is next to impossible when patients present with thoracic complications. This explains the higher incidence of abdominal and pelvic angiography in REBOA groups which we find to be an acceptable >tradeoff for accuracy in occlusion balloon positioning. We do not doubt that REBOA will be applicable as a bridge to definitive treatment in the ED, but indications and contraindications in the light of ACC must be further refined. Finally, the most critical point to remember is that any method inducing long-lasting ischemia to at least half or more of the body has serious potential to harm the patient. To this end, the decision to use REBOA or ACC should be part of a robust clinical governance framework in order to ensure high quality patient care and maximal survival chance [9].

### Limitations

Potential limitations of this study should be acknowledged. First, there remained some indication bias as previously discussed, indicating caution when interpreting results for clinical standpoints. However, we controlled for patient background using logistic regression and PS-matched analysis, when possible, and found two key points in this study. First, PS-matched analysis was one of the best methods for comparison because there was a relatively small sample size of survivors. Second, there was institutional bias although covariates were carefully selected on the basis of the assumption that none were affected directly by the intervention. This assumption could be a potential weakness and requires further study. With regard to mortality rates, a population-based study in England and Wales showed only major trauma centers had a high density of REBOA use and their rate was smaller than ours [9]. We, on the other hand, did not have institutional-level data, and therefore we could not control for it and this might account for our higher mortality rate. Although a potential weakness could be variability between physicians and institutions, AORTA registry data reveals that the general tendency of outcomes is the same [1]. Although selection bias may skew towards REBOA more than ACC in both AORTA and this study, we feel that our results are worth consideration to add to the scarce body of knowledge regarding this topic. Moreover, we did not have detailed data on REBOA or ACC such as the clamping time, the ballooning time, and the tactics of that therapy. Since the patients had the issue of ischemia/reperfusion injury, their outcome may have been influenced by time. A general assumption, however, is that clamping and ballooning times were kept as short as possible by the physicians because of the common knowledge that occlusion times should be kept to a minimum.

## Conclusions

Despite any residual indication bias, REBOA might be a favorable alternative method to ACC, especially for severe trauma below the diaphragm. Further study is needed to elucidate optimal indications.

## Key messages

- Patients who underwent AO had a high mortality.
- REBOA might be a favorable alternative method to ACC.

## Abbreviations

ACC: Aortic cross-clamping
AIS: Abbreviated Injury Scale
AO: Aortic occlusion
AORTA: Aortic Occlusion for Resuscitation in Trauma and Acute Care Surgery
CI: Confidence interval
ED: Emergency department
GCS: Glasgow Coma Scale
IQR: Interquartile range
ISS: Injury Severity Score
JTDB: Japan Trauma Data Bank
OR: Odds ratio
PS: Propensity score
REBOA: Resuscitative endovascular balloon occlusion of the aorta
RTS: Revised trauma score
SD: Standard deviation
TAE: Trans-catheter arterial embolization
TRISS: Trauma and injury severity score
