# Erratum to: Resuscitative endovascular balloon occlusion of the aorta versus aortic cross clamping among patients with critical trauma: a nationwide cohort study in Japan

**DOI:** 10.1186/s13054-017-1627-z

**Published:** 2017-02-22

**Authors:** Toshikazu Abe, Masatoshi Uchida, Isao Nagata, Daizoh Saitoh, Nanako Tamiya

**Affiliations:** 1 Department of Health Services Research, Faculty of Medicine, University of Tsukuba, 1-1-1, Tennodai, Tsukuba, 305-8577 Japan; 2 Department of Emergency Medicine, Tsukuba Medical Center Hospital, 1-3-1, Amakubo, Tsukuba, 305-8558 Japan; 3 Department of Traumatology and Emergency Medicine, National Defense Medical College, 3-2, Namiki, Tokorozawa, Saitama 359-8513 Japan

## Erratum

Following publication of the original article [1], it was brought to our attention that there were a few errors in Table 2:

**Table 2:** Outcome comparisons between REBOA and ACC

	REBOA (*n* = 636)	ACC (*n* = 267)	*P* value
Disposition at discharge			<0.001^*^
Died (in-hospital mortality)	405/607 (67%)	210/233 (90%)	
Transferred	118/607 (19%)	11/233 (4.7%)	
Home	83/607 (14%)	12/233 (5.2%)	
Other	1/607 (0.1%)	0/233 (0.0%)	
Disposition at ED			<0.001^*^
Died (ED mortality)	137/625 (22%)	130/264 (49%)	
ICU admission	472/625 (76%)	129/264 (49%)	
Ward admission	137/625 (22%)	4/264 (1.5%)	
Other	5/625 (1.8%)	1/264 (0.4%)	

The variables are shown as *n* (%)

*ACC* aortic cross clamping, *ED* emergency department, *ICU* intensive care unit, *REBOA* resuscitative endovascular balloon occlusion of the aorta

^*^Chi-square test

CU admission should read: ICU admission

11/233 (1.8%) should read: 11/233 (4.7%)

12/233 (2.0%) should read: 12/233 (5.2%)

The corrected table is presented in this erratum [Table 2].

Furthermore, the sentence “…only 14% (83/607) of REBOA patients and 2.0% (12/233) of ACC patients could leave the hospital and go home.” in the Discussion section should as a consequence read: “…only 14% (83/607) of REBOA patients and 5.2% (12/233) of ACC patients could leave the hospital and go home.”

This has now been corrected in this erratum.
